# Wild Carnivore Survey of *Echinococcus* Species in Slovenia

**DOI:** 10.3390/ani12172223

**Published:** 2022-08-29

**Authors:** Petra Bandelj, Rok Blagus, Gorazd Vengušt, Diana Žele Vengušt

**Affiliations:** 1 Veterinary Faculty, University of Ljubljana, Gerbičeva ulica 60, SI-1115 Ljubljana, Slovenia; 2 Institute for Biostatistics and Medical informatics, University of Ljubljana, Vrazov trg 2, SI-1104 Ljubljana, Slovenia; 3 Faculty of Sports, University of Ljubljana, Gortanova 22, SI-1000 Ljubljana, Slovenia; 4 Faculty of Mathematics, Natural Sciences and Information Technologies, University of Primorska, Glagoljaška ulica 8, SI-6000 Koper, Slovenia

**Keywords:** *Echinococcus multilocularis*, *Echinococcus granulosus sensu stricto*, *Echinococcus canadensis*, real time PCR, red fox, golden jackal, wildlife

## Abstract

**Simple Summary:**

Wild carnivores can harbor a dangerous tapeworm *Echinococcus* sp. that causes an important food-borne disease called echinococcosis. This study uses molecular methods to assess the prevalence of the tapeworm *Echinococcus multilocularis*, *E. canadensis,* and *E. granulosus sensu stricto* in the stool of red foxes, wolves, golden jackals, martens, lynxes, badgers, and otter. Red foxes (29.1%) and golden jackals (18%) were positive for *E. multilocularis* (EM), while all other animals were negative for all *Echinococcus* species tested in this study. Statistical analysis showed that the prevalence of EM is associated only with the region where the sample originated and not by species, age, or sex of the animal. Central and south regions of Slovenia have a higher EM prevalence and risk of infection. Due to habitat expansion and an increasing population, golden jackal may soon become an important source for human infection with EM.

**Abstract:**

Wild carnivores are definitive hosts and potential reservoirs for the *tapeworm Echinococcus* sp. which can cause cystic and alveolar echinococcosis. Both are considered neglected and important food-borne pandemics. This study is the first to molecularly test Slovenian wild carnivores for *Echinococcus* species that can cause disease in humans. Fecal samples from 210 red foxes (*Vulpes vulpes*), 42 wolves (*Canis lupus*), 39 golden jackals (*Canis aureus*), 18 martens (Marten sp.), 2 Eurasian lynx (*Lynx lynx*), 2 European badger (*Meles meles*), and 1 Eurasian otter (*Lutra lutra*) were examined for *Echinococcus granulosus sensu lato* (EGsl: *E. granulosus sensu stricto, E. canadensis*) and *E. multilocularis* (EM) using real-time PCR. Red foxes (29.1%) and golden jackals (18%) were positive for EM. All animals examined were negative for EGsl. Univariate analysis showed no significant differences in EM prevalence with respect to animal species (red fox vs., golden jackal) (*p* = 0.22), age (*p* = 0.12), and sex (*p* = 0.18). Prevalence of EM was associated with the region (*p* < 0.001), with regions in central and southern Slovenia having higher EM prevalence and risk of infection. Due to the increase in population and expansion of habitat, the golden jackal may soon become as important definitive host for EM as the red fox.

## 1. Introduction

Described already by the ancient Greek physicians [1,2], *Echinococcus* sp. is a life-threatening zoonotic parasite that is still relevant today. The small tapeworm has two obligate mammalian hosts, and its life cycle depends on predator–prey association [2]. The definitive host is always a carnivore, in which the adult tapeworm develops in the small intestine. When eggs are shed by the definitive host and ingested by the herbivorous or omnivorous intermediate host, the metacestode develops, usually in the viscera (liver, lungs) of the mammal [3]. Due to low host specificity, humans are accidental hosts for the larvae of *Echinococcus granulosus sensu latu* complex (EGsl) and *Echinococcus multilocularis* (EM), where it can cause cystic or alveolar echinococcosis, respectively [2,3]. Several genotypes have been described in the EGsl complex, namely *E. granulosus sensu stricto* (EGss) genotype G1 and G3, *E. equinus* (genotype G4), *E. ortleppi* (genotype G5), *E. canadensis* cluster (EC) (genotype G6-G8, G10), and *E. felidis* [2,4,5]. Cystic echinococcosis is predominantly caused by EGss, but infections with EC (G6 and G7) are also common [4,6]. Both cystic echinococcosis (CE) and alveolar echinococcosis (AE) are among the top four foodborne parasitosis in Europe [7,8]. *Echinococcus granulosus s.l.* has a more domestic life cycle (dog/domestic ungulate), but wild canids such as wolf (*Canis lupus*), golden jackal (*Canis aureus*), and red fox (*Vulpes vulpes*) also act as final hosts, that may pose a threat to humans or serve as a wildlife reservoir [9,10]. The sylvatic cycle (fox/rodent) is, however, more common for EM, where the main reservoir for the parasite is the red fox population. Other wild (wolf, golden jackal) and domestic (dog, cat) canids can be infected with EM, bringing the parasite closer to humans [9,10,11,12,13,14,15]. Unlike EGsl, which has a more global distribution, EM is mainly found in the northern hemisphere [8]. Several reports from European countries show a high prevalence of up to 62% in North-Eastern Europe [16] in the red fox population and almost 50% in Sweden and central Europe (France, Switzerland) [17]. Because EM prevalence can vary greatly from region to region within the same country, data must be carefully assessed to avoid its biased interpretation [13,16]. In Slovenia, a study of EM from the 2002–2005 red fox population revealed a relatively low prevalence of 2.6% [18]. *Echinococcus granulosus s.l.* has only been detected in intermediate hosts (pig, cattle) [19], and although its presence in the definitive hosts has not yet been studied, genotyping of human CE cases showed that EGss (G1-G3) and EC (G7) are present in the country [4].

The aim of this study is to evaluate the prevalence of EGsl and to reassess the prevalence of EM among wild carnivores in Slovenia using molecular methods to assess the regional distribution and risk of animal and human infection.

## 2. Materials and Methods

### 2.1. Samples

Fecal samples from 210 red foxes (*Vulpes vulpes*), 42 wolves (*Canis lupus*), and 39 golden jackals (*Canis aureus*) were collected at the Institute of Pathology, Wild Animals, Fish and Bees (Veterinary faculty, University of Ljubljana, Slovenia) along with 18 samples from martens (*Marten* sp.), two samples each from Eurasian lynx (*Lynx lynx*) and European badger (*Meles meles*), and one sample from Eurasian otter (*Lutra lutra*). The carcasses were collected as part of the regular annual hunting bag throughout the Slovenian territory. Samples from red foxes were collected between 2019 and 2022, while samples from wolves, golden jackals, and other animals were collected over a ten-year period. All samples were stored at –80 °C for at least one month prior to processing and DNA isolation to avoid contamination with viable *Echinococcus* eggs.

All samples were collected postmortem, so the ethics committee/welfare authority approval was not required.

### 2.2. Methods

#### 2.2.1. Age Determination

The age of older animals (one year and older) was determined by counting the increment layers of the secondary dental cementum of a cut lower canine root and, in young animals, by the size of pulp cavity according to Roulichova and Andera [20]. The age of wolves was determined by counting the cementum layers on cross-sections of the first premolar in the Matson laboratory (Missoula, MT, USA).

#### 2.2.2. Molecular Methods

Fecal DNA isolation was performed using a protocol from the SmartHelix™ First DNAid kit (IFB, Ljubljana, Slovenia) as previously described [21]. Three different published real time polymerase chain reactions (separately fPCR) were used for the detection of EM (G5), EGss (G1-G3), and EC (G6/G7). *Echinococcus multilocularis* was detected using a protocol by Knapp et al. [22] with a detection limit of 5 × 10^−5^ ng/µL, corresponding to one EM egg. For EGss, a protocol by Maksimov et al. [23] was used, while EC was screened using a protocol published by Grech-Angelini et al. [24]. All qPCRs were performed in a 96-well plate format using ABI 7500 Fast (Applied Biosystems^®^, Waltham, MA, USA) with the same thermal cycling conditions consisting of a preheating step at 50 °C for 2 min, followed by 95 °C for 10 min and 45 cycles of denaturation at 95 °C for 15 s with annealing and extension at 60 °C for 1 min. When optimizing the protocols, the optimal total DNA/PCR mix volume was set at 2/20 μL. Fast Start Universal Probe Master (Rox; Roche^®^, Basel, Swizerland) was used for the EM and EG protocols, whereas Maxima Probe qPCR Master Mix (Thermo Scientific^®^, Waltham, MA, USA) was used for the EC protocol. The EC protocol showed marked inhibition when Fast Start Universal Probe Master (Rox; Roche^®^) or TaqMan Universal Master Mix (Applied Biosystems^®^) were used to detect EC DNA in clinical samples, whereas no inhibition was detected when Maxima Probe qPCR Master Mix (Thermo Scientific^®^) was used. All samples were tested twice, and DNA from negative samples was diluted (1:10) and retested to rule out possible inhibition. Inhibition of the PCR is common in complex clinical samples, such as feces, where there is excess DNA that can interfere/inhibit with the amplification of the targeted DNA [25]. No additional positive samples were found when DNA was retested and diluted. The limit of detection for EM was 4.4 × 10^−5^ ng/µL, confirming the detection limit established by Knapp et al [22]. The detection limit was 2.44 × 10^−2^ ng/µL for EGss and 3.2 × 10^−5^ ng/µL for EC. Positive reference samples for EM, Egss and EC were provided by the European Union Reference Laboratory for Parasites (Istituto Superiore di Sanità, Rome, Italy) (Table 1).

#### 2.2.3. Statistical Analysis

Data were summarized as frequencies (%). Univariate associations between EM prevalence (positive vs. negative) and sex (male, female), age (3 categories: 0 years, 1 year, ≥2 years), and region (11 regions) (Table 2) were tested using chi-square test with Yates continuity correction. The multivariate analysis was performed using a binary logistic regression. Random intercept by region was included in the model to account for potential effect of the region (due to the large number of regions, the region could not be considered as a fixed effect). Results are presented as conditional odds ratios (Ors) with corresponding 95% confidence intervals (Cis). Effects were considered significant when the *p*-value was lower than 0.05. R language for statistical computing (R version 3.6.1) was used for the analyses [26]. R package lme4 was used to fit the model using 10 points per axis for evaluating the Gauss–Hermite approximation to the log-likelihood.

## 3. Results

*Echinococcus multilocularis* DNA was found in the feces of 61/210 (29.1%; CI 0.23–0.36) red foxes and 7/39 (18%; CI 0.07–0.34) golden jackals (Table 2). Wolves (*n* = 42), martens (*n* = 18), Eurasian lynx (*n* = 2), European badger (*n* = 2), and Eurasian otter (*n* = 1) were negative for the presence of EM. All samples were negative for EGss genotype G1-G3 and EC genotype G6 and G7.

**Figure 1:**
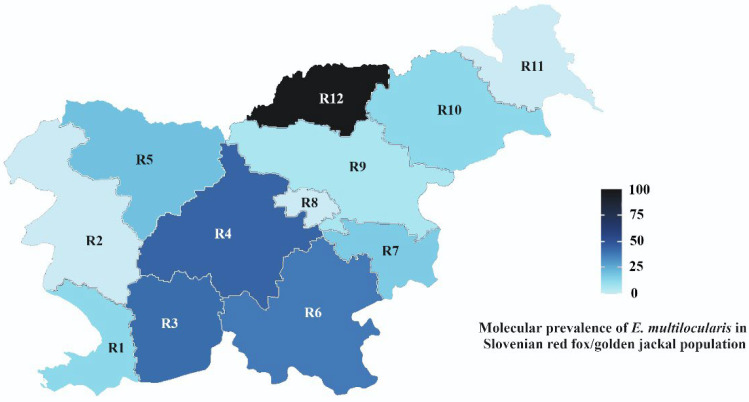
Molecular prevalence of *E. multilocularis* in the Slovenian red fox/golden jackal population (%). Regions (R): R1—obalno kraska, R2—goriska, R3—primorsko notranjska, R4—osrednjeslovenska, R5—gorenjska, R6— jugovzhodna Slovenija, R7—posavska, R8—zasavska, R9—savinjska, R10—podravska, R11—pomurska, R12—koroska.

Univariate analysis revealed no statistically significant association between the prevalence of EM and animal species (red fox and golden jackal) (*p* = 0.22), sex (*p* = 0.18), and age groups (*p* = 0.12) (Table 2). However, sampling location was highly significant (p < 0.001) (Table 2). Regions R3 (primorsko notranjska), R4 (osrednjeslovenska), and R6 (jugovzhodna Slovenia) had a prevalence of 37.9% to 42.2% in the red fox/golden jackal population (Table 2, Figure 1). Region R12 (koroska) had an EM prevalence of 100%, but only two samples were collected and tested from this region. 

In the multivariate analysis, when adjusted for region, no statistically significant association for an EM positive outcome was observed; species: OR 0.57 (95% CI: 0.2–1.57), sex: OR 1.77 (95% CI: 0.91–3.44), and age: 1 year vs. 0 years, OR 0.91 (95% CI: 0.42–1.92); ≥2 years vs. 1 year, OR 1.75 (95% CI: 0.79–3.87) (Table 3).

## 4. Discussion

Wild carnivores are definitive hosts and potential reservoirs for the tapeworm *Echinococcus* sp. which can cause severe disease in animals and humans [27,28]. Cystic and alveolar echinococcosis, caused by EGsl and EM, respectively, are recognized as neglected and important food-borne pandemics [29,30]. This study is the first to address the need for molecular testing of Slovenian wild carnivores for the presence of European *Echinococcus* species capable of causing disease in humans. 

The EGss and EC are part of the EGsl complex and have been recognized as the cause of disease in several patients in Slovenia [4]. Sporadic cases of cystic echinococcosis were reported in intermediate hosts, such as pigs and cattle [19], but no surveys were conducted in wild or domestic animals in Slovenia. *Echinococcus granulosus s.s.* and EC (G6/G7) have adapted to a domestic life cycle over the years. Their life cycle in the wildlife (wild sheep, wild goat, cervids, wild boar/wolves, jackals, foxes) is considered more ancestral [28,31,32]. However, due to the increase in wild prey-predator populations susceptible to the parasite, sylvatic transmission is predicted to regain at least some of its former role in Europe [28,32]. *Echinococcus granulosus* (genotype 1) was found in a jackal and a wolf from Bulgaria [28,33] and the occurrence of EGsl in Iberian wolves was 1.5% in Portugal [34] and 5.6–15% in the Italian Northern Apennine population of wolves [35,36]. Red foxes in Great Britain and Corsica (France) served as a definite host for EGsl when access to sheep or pigs’ carcasses was available [28]. However, our study showed zero prevalence of EGss and EC in wolves, golden jackals, red foxes, martens, lynxes, badgers, and otter, suggesting that the EGsl parasite is still mainly maintained in the domestic life cycle in Slovenia. Further studies are needed to assess the prevalence of the parasite in domestic definite and intermediate hosts. In Slovenia, sustainable livestock production in mountainous/hilly perennial grasslands with shallow soil of poor quality (Dinaric Karst area) is mainly limited to sheep and goat farming, which is also the main Slovenian habitat for wolves [37]. Small ruminants are usually pastured near forest edges, where wolves reside, further increasing the risk of attacks on livestock. The wolf density in Slovenia of 1 wolf/100 km^2^ is considered low, but as the population increases, the number of attacks on sheep also increases [37]. Domestic livestock represents 10% of the wolves’ diet [38], which could serve as an entry point for a possible reinstation of EGsl from the domestic to a semi-domestic and sylvatic life cycle. This could reinforce the presence of EGsl on Slovenian territory as has done in Italy [35].

While this study is the first to assess the occurrence of EGsl in animals from Slovenia, EM was previously reported in the 2002–2005 in the red fox population with a prevalence of 2.6% [18]. In this study, a prevalence of 29.1% was found in Slovenian red foxes sampled between 2019 and 2022, which is similar to the reported pooled prevalence in other countries with high occurrence of EM in Central and North-Eastern Europe [16]. Based on the results of this study, Slovenia has moved from a low prevalence group [16] to a high prevalence group in less than two decades. The reasons for the high prevalence of EM in red foxes; could be due to a successful rabies vaccination campaign, the subsequent increase in their population [39], and the introduction of a new predator, the golden jackal. They have been sporadically sighted in Slovenia since 1952, but permanent territorial jackal families were first reported in Central Slovenia (Region 4 in this study) in 2008 [40]. Since then, they have spread throughout the country and into Central and Eastern Europe [41]. Golden jackals are susceptible to *Echinococcus* species and may serve as the second definitive hosts, contributing to an increase in the overall prevalence of EM [10,16,42,43]. A report from Serbia suggests a prevalence of EM in golden jackals of 14.3% [44], which is only slightly lower than the 18% prevalence found in the Slovenian jackal population from this study. In our study, the prevalence of EM did not differ between animal species (red fox vs. golden jackal), which is consistent with previous published studies from Hungary and Serbia [28,44,45]. This suggests that red fox and golden jackal have similar feeding behavior, with the main prey being small mammals (rodents), while wolves in Slovenia feed mainly on cervids, wild boar, and small domestic ruminants [28,37,38,44]. The pooled prevalence in the European wolf population is 1.4% [16], so it is not surprising, that no wolf in this study tested positive for EM. All other animals tested (lynx, otter, martens, badgers) from this study were negative for EM, which was expected based on other published studies [16,28]. Overall, only one lynx from Turkey [46], stone and pine martens from the European part of Russia [28] were found to be positive for EM. However, more samples should be tested for EM to properly assess the prevalence of EM in these species. The dramatic increase in EM prevalence observed in red foxes and jackals in Slovenia, combined with the zoonotic potential of the parasite, warrants a more vigilant approach even in species that are not considered primary hosts for the tapeworm. 

In the red fox and golden jackal populations from our study, no association was found between EM prevalence and age, and sex. This is consistent with previously published studies [47,48,49]. However, the prevalence of EM was associated with region where the sampled animals were collected. Thus, EM prevalence in Slovenia ranged from 0 (R2 – goriska, R11 – pomurska) to 42.19% (R4 – osrednjeslovenska) and 100% in region R12 (koroska). However, only two foxes were sampled in region R12, which cannot represent a true red fox population EM prevalence in this region. As shown previously, studies that focus on limited areas within a country and are not accompanied by other studies from different regions, may not show the true EM prevalence at the national level [16,28]. Accordingly, 11 of the 12 regions in Slovenia were included in this study. The only region that did not have a sample was region R8 (Zasavska), which is also the smallest region in Slovenia [50]. Region R4 (osrednjeslovenska) had a high EM prevalence of 42.19% and is also the region with the highest population density of 228 person/km^2^ in Slovenia [51]. Several studies have found a higher prevalence of EM positive foxes in an urban versus a rural environment and referred to it as EM urbanization [39,52]. On the national level this study shows a more complete assessment of the EM prevalence in definitive sylvatic hosts and a distinct prevalence in central and South regions of Slovenia.

## 5. Conclusions

In conclusion, this is the first study in which molecular methods were used to assess EGsl and EM in Slovenian wildlife. Although no animal was positive for EGsl, the prevalence of EM in the red fox population increased dramatically since 2002–2005 [18]. This study is also the first to report EM positive golden jackals in Slovenia. Their EM prevalence and habitat expansion suggest that they may soon be considered as important definitive host as the red fox. Both species are well acclimated to urban environments and come close to human residencies [52]. It is inevitable that humans and their pets (dogs, cats) will come in contact with EM in a contaminated environment or through an infected intermediate host. Based on the results of this study, regions in central and southern Slovenia should be considered at higher risk for human and pet exposure. Further studies are needed to determine the risk factors associated with the high prevalence of EM and the prevalence of *Echinococcus* sp. in intermediate hosts, and to evaluate the domestic cycles of EGsl and EM. 

## Figures and Tables

**Table 1:** Description of the different primers and probes used in three separate qPCR to detect *E. multilocularis* (EM), *E. granulosus s.s.* (Egss) and *E. canadensis* (EC).

Assay Name (Gene Targeted)	Primer/ Probe	Oligonucleotide Sequences (5′-3′)	Product Size	Reference
EM (rrnL)	Forward	CTGTGATCTTGGTGTAGTAGTTGAGATTT	(bp) 84	[22]
	Reverse	GGCTTACGCCGGTCTTAACTC		
	Probe	FAM -TGGTCTGTTCGACCTTTTTAGCCTCCAT – TAMRA		
Egss (cox1)	Forward	AGGGGCTGGTGTTGGTTGGA	80	[23]
	Reverse	TGAAACACCAGCCAAATGCAGAGA		
	Probe	FAM – TCCGCCGTTGTCCTCGTCGT – BHQ1		
EC (nad5)	Forward	TCTTTCTGATAGACGAGGTTAGG	109	[24]
	Reverse	TCCATAAAGCCAAAAATTGTAC		
	Probe	Cy5 – CGGTGGTTTGTAGTGTGAGTTTGGTG – BHQ2		

**Table 2:** Prevalence of *E. multilocularis* in red fox and golden jackal populations by species, sex, age, and region.

.	Tested Animals		EM Positive (%)			*p* *
Species						0.22
Red fox	210		61 (29.1)			
Golden jackal	39		7 (18)			
	Red fox	Golden jackal	Red fox	Golden jackal	Red fox + Golden jackal	
Sex						0.18
male	133	28	43 (32.3)	6 (21.4)	49/161 (30.4)	
female	77	11	18 (23.4)	1 (9.1)	19/88 (21.6)	
Age (years)						0.12
juvenile (0)	57	6	19 (33.3)	0	19/63 (30.2)	
young adult (1)	108	30	24 (22.2)	7 (23.3)	31/138 (22.5)	
adult (≥2)	45	3	18 (40)	0	18/48 (37.5)	
Region						<0.001
R1 obalno kraska	2	8	0	1 (12.5)	1/10 (10)	
R2 goriska	8	2	0	0	0/10 (0)	
R3 primorsko notranjska	12	3	6 (50)	0	6/15 (40)	
R4 osrednjeslovenska	51	13	24 (47.1)	3 (23.1)	27/64 (42.2)	
R5 gorenjska	9	2	1 (11.1)	1 (50)	2/11 (18.2)	
R6 jugovzhodna slovenija	66	/	25 (37.9)	/	25/66 (37.9)	
R7 posavska	11	9	1 (9.1)	2 (22.2)	3/20 (15)	
R8 zasavska	/	/	/	/	/	
R9 savinjska	30	2	1 (3.3)	/	1/32 (3.1)	
R10 podravska	9	/	1 (11.1)	/	1/9 (11.1)	
R11 pomurska	10	/	0	/	0/10 (0)	
R12 koroska	2	/	2 (100)	/	2/2 (100)	

Data are frequencies (%), * *p*-value from a chi-squared test with continuity correction.

**Table 3:** Risk factors for prevalence of *E. multilocularis* in red fox and golden jackal. Results are conditional odds ratio (OR), 95% confidence intervals (CI), and *p*-values (model area under the curve 0.77).

Red Fox/ Golden Jackal.	OR	95% CI	*p*-Values
Species (red fox vs. golden jackal)	0.57	0.2–1.57	0.28
Sex (male vs. female)	1.77	0.91–3.44	0.1
Age (y)			0.37
1 vs. 0	0.91	0.42–1.92	0.8
≥2 vs. 1	1.75	0.79–3.87	0.17

## Data Availability

The data presented in this study are available on request from the corresponding author.
